# The Art of Not Hearing

**Published:** 1897-04

**Authors:** 


### The Art of not Hearing.

The art of not hearing should be learned by all. There are so many things which it is painful to hear, very many which, if heard, will disturb the temper, corrupt simplicity and modesty, detract from contentment and happiness. If a person falls into a violent passion and calls all manner of names, at the first word we should shut our ears and hear no more. If in a quiet voyage of life we find ourselves caught in one of those domestic whirlwinds of scolding, we should shut our ears as a sailor would furl his sail, and, making all tight, scud before the gale. If a hot, restless man begins to inflame our feelings, we should consider what mischief the fiery sparks may do in our magazine below, where our temper is kept, and instantly close the door. If all the petty things said of a man by heedless and ill-natured idlers were brought home to him, he would become a mere walking pin-cushion stuck full of sharp remarks. If we would be happy when among good men, we should open our ears; when among bad men, shut them. It is not worth while to hear what our neighbors say about our children, what our rivals say about our business, our dress, or our affairs.—*Pacific Record of Medicine and Surgery.*

